# Risk of Dementia Among Patients With Diabetes in a Multidisciplinary, Primary Care Management Program

**DOI:** 10.1001/jamanetworkopen.2023.55733

**Published:** 2024-02-12

**Authors:** Kailu Wang, Shi Zhao, Eric Kam-Pui Lee, Susan Zi-May Yau, Yushan Wu, Chi-Tim Hung, Eng-Kiong Yeoh

**Affiliations:** 1 Centre for Health Systems and Policy Research, Jockey Club School of Public Health and Primary Care, Faculty of Medicine, The Chinese University of Hong Kong, Hong Kong, China; 2 School of Public Health, Tianjin Medical University, Tianjin, China; 3 Tianjin Key Laboratory of Environment, Nutrition, and Public Health, Tianjin Medical University, Tianjin, China; 4 Jockey Club School of Public Health and Primary Care, Faculty of Medicine, The Chinese University of Hong Kong, Hong Kong, China

## Abstract

**Importance:**

Although poorly controlled diabetes is associated with a higher incidence of dementia, few studies have examined the association of diabetes management interventions with dementia incidence.

**Objective:**

To examine the association of receiving a multidisciplinary diabetes management program (the Risk Assessment and Management Program-Diabetes Mellitus [RAMP-DM]) that enables better glycemic control with subsequent risk of dementia incidence and the association of dementia with glycemic control.

**Design, Setting, and Participants:**

This territory-wide, retrospective, matched cohort study with more than 8 years of follow-up was conducted using electronic health care records from all the patients who used public health care services in Hong Kong from 2011 to 2019. Eligible participants included all patients with type 2 diabetes (T2D) who were managed in primary care settings. Patients who received RAMP-DM were matched in a 1:1 ratio with patients who received usual care only. Data analysis occurred from April 2023 to July 2023.

**Exposures:**

Diagnosis of T2D, hemoglobin A_1C_ (HbA_1C_) level, and attendance at a general outpatient clinic or family medicine clinic. Patients received either RAMP-DM or usual care.

**Main Outcomes and Measures:**

Incidence of all-cause dementia and subtypes of dementia were compared between the RAMP-DM and usual care participants using a Cox proportional hazard model with other baseline characteristics, biomarkers, and medication history adjusted. HbA_1C_ levels were measured as a secondary outcome.

**Results:**

Among the 55 618 matched participants (mean [SD] age, 62.28 [11.90] years; 28 561 female [51.4%]; 27 057 male [48.6%]), including the 27 809 patients in the RAMP-DM group and 27 809 patients in the usual care group, patients had been diagnosed with T2D for a mean (SD) of 5.90 (4.20) years. During a median (IQR) follow-up period of 8.4 (6.8-8.8) years, 1938 patients in the RAMP-DM group (6.97%) and 2728 patients in the usual care group (9.81%) received a diagnosis of dementia. Compared with those receiving usual care, RAMP-DM participants had a lower risk of developing all-cause dementia (adjusted hazard ratio [aHR], 0.72; 95% CI, 0.68-0.77; *P* < .001), Alzheimer disease (aHR, 0.85; 95% CI, 0.76-0.96; *P* = .009), vascular dementia (aHR, 0.61; 95% CI, 0.51-0.73; *P* < .001), and other or unspecified dementia (aHR, 0.71; 95% CI, 0.66-0.77; *P* < .001). Compared with having a mean HbA_1C_ level during the first 3 years after cohort entry between 6.5% and 7.5%, a higher risk of dementia incidence was detected for patients with a 3-year mean HbA_1C _level greater than 8.5% (aHR, 1.54; 95% CI, 1.31-1.80]), between 7.5% and 8.5% (aHR, 1.33; 95% CI, 1.19-1.48), between 6% and 6.5% (aHR, 1.17; 95% CI, 1.07-1.29), and 6% or less (aHR, 1.39; 95% CI, 1.24-1.57).

**Conclusions and Relevance:**

In this cohort study of patients with T2D, the findings strengthened evidence of an association of glycemic control with dementia incidence, and revealed that a multidisciplinary primary care diabetes management program was associated with beneficial outcomes for T2D patients against dementia and its major subtypes. A moderate glycemic control target of HbA_1C_ between 6.5% and 7.5% was associated with lower dementia incidence.

## Key Points

**Question:**

Is a multidisciplinary diabetes management program in primary care settings associated with a reduced risk of dementia incidence among patients with type 2 diabetes (T2D)?

**Findings:**

This cohort study of 55 618 participants in Hong Kong found patients with T2D who attended the diabetes primary care management program had a 28% lower risk of all-cause dementia incidence compared with patients who did not attend the program and received only usual care.

**Meaning:**

These findings suggest that a multidisciplinary diabetes management program in primary care settings was associated with beneficial outcomes for patients with T2D against dementia.

## Teaser

This cohort study analayzes the association of a multidisciplinary diabetes management program with incident dementia among patients with type 2 diabetes in Hong Kong.

## Introduction

Dementia poses a substantial disease burden on global population health and continues to increase in incidence with an aging population worldwide.^1^ The risk of dementia incidence is higher among patients with type 2 diabetes (T2D),^2^ which was 1 of the top 10 diseases contributing to global disability-adjusted life years from 1990 to 2019, increasing throughout this period, especially in older adults.^3^ Several systematic reviews^4,5^ have summarized that T2D is associated with approximately 50% greater likelihood of all-cause dementia, and there is a higher risk in Asian populations than in other populations. T2D is associated with the presence of some pathological features of Alzheimer disease (AD) and other dementias, including vascular damage and associated pathways^6^; overaccumulation of amyloid-β and tau phosphorylation^7,8,9^; and chronic neuroinflammation, insulin resistance, and blood-brain barrier damage that result from hyperglycemia and hyperinsulinemia.^7,10,11^

Although a higher hemoglobin A_1C_ (HbA_1C_) level is associated with a greater risk of dementia incidence among patients with T2D,^12,13,14,15^ the effectiveness of glycemic control interventions to reduce dementia incidence remains uncertain. To facilitate better management of T2D, management programs involving multidisciplinary teams that consist of primary care clinicians, specialists, nurses, and allied health professionals have been implemented in many countries.^16,17,18,19,20^ Although randomized clinical trials (RCTs) have confirmed the effectiveness of such multidisciplinary management on mortality and diabetic complications,^16,19,20,21^ only 1 trial^22^ evaluated the cognitive outcomes of patients with T2D. That study^22^ revealed that intensive glucose lowering was associated with higher total brain volume, but did not significantly enhance cognitive performance in a 40-month follow-up.^22^ Nevertheless, further studies that assess the association of diabetes management with dementia outcomes are needed, particularly those with a longer follow-up period and more individualized glucose control targets.

In Hong Kong, more than 90% of patients with T2D are managed within its public health care system.^23^ A protocol-driven, multidisciplinary, primary care management program called Risk Assessment and Management Program-Diabetes Mellitus (RAMP-DM) was launched in 2009 to provide cardiovascular risk assessment, enable early detection of complications, and enhance diabetes management. RAMP-DM has been found to improve glycemic control and reduce the risk of mortality by 55%, macrovascular events by 48%, and microvascular events by 32%, with a follow-up period greater than 9 years.^24^ In light of the association of glycemic control with dementia risk, the current study therefore aimed to examine the association of using RAMP-DM services with the risk of all-cause dementia incidence and subtypes of dementia incidence. We further examined the association of glycemic control with the incidence of dementia. Our findings may provide insights into the prevention of dementia among patients with T2D.

## Methods

This cohort study was approved by the Survey and Behavioral Research Ethics committee of the Chinese University of Hong Kong with a waiver of informed consent because only secondary anonymized data were used in accordance with the Common Rule. This study was reported following the Strengthening the Reporting of Observational Studies in Epidemiology (STROBE) reporting guideline.

### RAMP-DM Services and Usual Care

Since the implementation of RAMP-DM in late 2009, patients with T2D who attended publicly funded outpatient clinics were invited to join this program on a voluntary basis. Patients who did not join RAMP-DM immediately upon invitation had the option to join later. RAMP-DM was provided by a multidisciplinary team (including nurses, doctors, and allied health professionals), with nurses taking the lead role in primary care with reference to the Joint Asia Diabetes Evaluation model.^25^ The workflow of RAMP-DM has been published in a previous study.^26^ Patients who joined the RAMP-DM program first received comprehensive assessments on lifestyle behaviors and drug adherence, screening for T2D complications (by physical examination, laboratory testing, and retinal photography), and estimation of cardiovascular risks at program intake. Health education and general guidance for diabetes management were also provided by nurses to all participants. Risk factors assessed included smoking status, obesity, hypertension, hyperlipidemia, diabetic retinopathy, albuminuria, foot problems, and estimated glomerular filtration rate. Based on the aforementioned risk factors, patients were stratified into different risk levels and referred to primary care physicians at general outpatient clinics (GOPCs), specialists, and/or allied health services (eg, a dietitian) according to their assessed risk levels and HbA_1C_ levels (ie, HbA_1C_ <7% or <8.5% [to convert HbA1c to proportion of total hemoglobin, multiply by 0.01]). Patients with HbA_1C_ levels of 7% or greater and higher risk level would receive more intensified interventions and more frequent follow-ups by nurses, general physicians, or specialists. RAMP-DM assessment was repeated continuously once every 1 to 3 years based on estimated risk level, and the patients would be referred to a specialist for related diabetic complications.

Usual care was provided to all patients with T2D at GOPCs regardless of whether they joined RAMP-DM or not, and they were seen and managed by their primary care physicians every 2 to 4 months. Their physicians conducted physical examinations, arranged investigations, prescribed and adjusted medications, and referred patients to specialists as deemed necessary. Consultations by nurses and allied health professionals were arranged on a referral basis based on doctor’s assessment of patients.

### Study Participants

This retrospective cohort study with more than an 8-year follow-up period was conducted from 2011 to 2019 and used the electronic health records (EHRs) collected from the public health care system in Hong Kong. Adult patients with a T2D diagnosis who attended GOPCs or family medicine clinics in 2011 were identified. T2D was ascertained using the *International Statistical Classification of Diseases and Related Health Problems, Tenth Revision (ICD-10)* code E11^27^ or the *International Classification of Primary Care, Second Edition* (*ICPC-2*) code T90. Patients with type 1 diabetes, gestational diabetes, or any type of dementia prior to the index date were excluded to engage patients with only T2D and who were free of outcome events at the index date (codes listed in eTable 1 in Supplement 1). Those without any public health care service records during follow-up (considered as loss of follow-up) were also excluded. The patients who attended RAMP-DM services plus usual care since 2011 were classified into the RAMP-DM group (treatment group), while those who had never joined RAMP-DM during the period on or before December 31, 2019 (ie, the end of the study period due to data availability at the time of study), were classified into the usual care group (control group). The patients who joined RAMP-DM services between 2012 and 2019 were not included in the study because there may not have been sufficient follow-up time from the time of joining RAMP-DM to observe the incidence of dementia as the outcome. The index date was defined as the day when intake assessment was received for the RAMP-DM group or the day of first attendance at a GOPC or family medicine clinic for diabetes follow-up since January 2011 for the usual care group. The follow-up periods ended upon the occurrence of the outcome event, death, or the end of study period (December 2019), whichever came earlier.

### Data Source

The data were retrieved from the Hospital Authority Data Collaboration Laboratory (HADCL) of the Hospital Authority and Hong Kong Special Administrative Region of China, which covers all the EHRs of over 9 million individuals who used public health care services from 2000 to 2019. Mortality information of the individuals was obtained through HADCL from the death registry of Hong Kong, including all known deaths within and outside of the public hospitals.

### Outcome and Exposure Ascertainment

The primary outcome event was the incidence of dementia, which was determined based on *ICD-10 *codes (UK-Biobank definitions can be found in eTable 1 in Supplement 1) or *ICPC-2* codes (P70) that indicate diagnosis of any type of dementia, and the use of medications prescribed for dementia (donepezil, galantamine, and rivastigmine).^28^ The identification of dementia prior to the index date also adopted this criteria. Diagnosis of dementia was made in the usual practice of clinical teams based on clinical assessments and investigations with reference to the *Diagnostic and Statistical Manual of Mental Disorders* (Fourth Edition) and *Diagnostic and Statistical Manual of Mental Disorders* (Fifth Edition). Participants with dementia caused by alcohol, drugs, or infectious agents were excluded. The day of dementia diagnosis or the day of the initial medication prescription was considered as the date of the outcome event. The secondary outcomes included the incidence of AD, vascular dementia (VD), and other type of dementia (including unspecified dementias).

In the primary analysis, the exposure factor was the use of RAMP-DM. As one of the main primary care targets of the RAMP-DM program, the management of HbA_1C_ levels of patients with T2D was monitored on a regular basis after program enrollment.^25^ As such, we explored the association of HbA_1C_ levels at the early stage after joining RAMP-DM with the incidence of dementia. In our supplementary analysis, the mean level of HbA_1C_ between the first and third year after the index date was used as an exposure factor among patients in the RAMP-DM group, while the usual care group was excluded from the supplemental analysis. This time interval for calculating mean HbA_1C_ levels was selected because RAMP-DM participants received assessments for their diabetes management every 3 years at maximum as previously indicated. The HbA_1C_ levels were was categorized into 5 groups (≤6.0%; 6.0%-6.5%; 6.5%-7.5%; 7.5%-8.5%; and >8.5%).^29^ The outcomes were defined as the incidence of dementia as indicated above. The follow-up period included the time period during the entire follow-up period after the index date and 3 years after the index date in 2 separate analyses.

### Covariates

The following covariates at baseline (ie, index date) were retrieved: age at baseline; sex; recipient of public assistance; living in an elderly care home; duration since diagnosis of T2D; diagnosis of hypertension; Charlson Comorbidity Index (CCI); any hypoglycemic, macrovascular, or microvascular events; use of services in RAMP for patients with hypertension; HbA_1C_ level; fasting glucose level; body mass index (BMI; calculated as weight in kilograms divided by height in meters squared); systolic and diastolic blood pressure; low-density lipoprotein cholesterol level; total cholesterol to high-density lipoprotein cholesterol ratio; triglycerides; estimated glomerular filtration rate; and use of insulin, oral antidiabetic (including metformin, glipizide, and others), antihypertensive, and lipid-lowering drugs.^13,24,28^

### Propensity Score Matching

The propensity score was estimated by a multivariate logistic regression model. In this study, participants with T2D who received RAMP-DM services were matched to a pool of participants who received usual care only. Patients with missing baseline characteristics were excluded. A comparison of baseline characteristics between the patients with and without missing values is presented in eTable 2 in Supplement 1. A matching ratio of 1:1 was practiced using the nearest-neighbor approach with discard for both groups and a caliper at 0.2-fold the SD, where participants were matched for baseline covariates, including sociodemographical factors, clinical characteristics, laboratory testing outcomes, and prescription records of medications. These variables, as shown in the previous section, were chosen based on possible or known associations with the risk of T2D progression to reduce the likelihood of selection bias from the intention to receive RAMP-DM services and its association with progression risk of T2D (including our primary outcome, dementia), and, hence, facilitated the comparison between the risk of dementia onset across 2 types of intervention groups.

For each variable, the postmatching absolute standardized mean difference (ASMD) between the RAMP-DM group and the usual care group was calculated. An ASMD less than 0.1 was considered to be a satisfactory balance of the baseline conditions between the 2 groups.

### Statistical Analysis

The baseline characteristics were described and compared between the RAMP-DM group and the usual care group (reference). To compare the outcomes between the groups, the cumulative incidence rate and incidence rate per person-year were estimated. The crude absolute risk reduction (ARR) and relative risk reduction (RRR) were calculated for both groups after matching. The time to incidence of dementia was visualized using a Kaplan-Meier curve. A multivariate Cox proportional hazard model was applied to calculate the difference in the hazard rates of dementia in the 2 groups, and the adjusted hazard ratio (aHR) of the RAMP-DM group was estimated relative to the usual care group. A 2-sided Wald test was performed to calculate the *P* values of the aHR estimates as a measurement of statistical significance level, where statistical significance was defined as *P*  < .05. The trends of HbA_1C_ levels of both groups throughout the follow-up period were also described.

Subgroup analysis on the Cox model was performed for sex, public assistance, elderly home residency, hypertension diagnosis, HbA_1C_ level (≤6.5%; 6.5%-7.5%; and >7.5%), and CCI score (0; 1-2; and ≥3). Interactions between these subgroup factors and the use of RAMP-DM were tested to ascertain whether differences in the levels of association were found across these subgroups. In the supplemental analysis, a Cox proportional hazard regression model was used to examine the relative risk of dementia incidence among patients with different HbA_1C_ levels in their first through third years after cohort entry, with adjustment of the same set of covariates used for propensity score matching.

Sensitivity analysis was performed using the Cox model for (1) all complete cases without matching; (2) the inclusion of extra covariates including outpatient service use and emergency department visits in the preceding year, hospitalizations in the preceding 3 years, and the presence of hyperglycemic events (diabetic ketoacidosis and hyperosmolar hyperglycemic state) prior to index date in the multiple Cox proportional hazard model; (3) excluding patients with dementia diagnosis or death within 2 years after cohort entry to avoid potential reverse causality where patients with dementia or worse health conditions may be less likely to join RAMP-DM in addition to usual care; (4) patients older than 60 years; (5) excluding patients who died during the follow-up period to avoid death as a competing outcome; and (6) excluding patients younger than 80 years old who died during follow-up.

All data processing and statistical analyses were carried out by using R statistical software version 4.1.1 (R Project for Statistical Computing). Data analysis was conducted from April 2023 to July 2023.

## Results

The 73 280 patients who joined RAMP-DM in 2011 (RAMP-DM group) and the 32 014 patients who did not join RAMP-DM from 2011 to December 2019 (usual care group) were identified based on the inclusion and exclusion criteria (eFigure 1 in Supplement 1). Comparison of baseline characteristics between patients with and without missing values can be found in eTable 2 in Supplement 1. After propensity score matching, a total of 55 618 patients were included (mean [SD] age, 68.28 [11.90] years; 28 561 female [51.4%]; 27 057 male [48.6%]) with 27 809 patients in the RAMP-DM group (median [IQR] age, 69 [60-77] years) and 27 809 patients in the usual care group (median [IQR] age, 70 [59-78] years) (Table 1). At baseline, 9119 participants (16.4%) received public assistance and 805 (1.4%) lived in an elderly home. The patients had been diagnosed with T2D for a mean (SD) of 5.90 (4.20) years and their mean (SD) HbA_1C_ level was 7.31% (1.37%). ASMDs of all baseline variables were less than 0.100 and lower than those in unmatched patients.

**Table 1.** Sample Characteristics for Unmatched and Propensity Score–Matched Patients

Characteristic	Unmatched cohort, No. (%)			ASMD	Matched cohort, No. (%)			ASMD
	Overall (N = 105 294)	RAMP-DM group (n =73 280)	Usual care group (n = 32 014)		Overall (N = 55 618)	RAMP-DM group (n = 27 809)	Usual care group (n = 27 809)	
Sociodemographic								
Age at baseline, mean (SD) y	66.13 (11.68)	64.64 (11.26)	69.54 (12.57)	0.390^a^	68.28 (11.90)	68.04 (11.14)	68.52 (12.61)	0.038
Sex								
Female	55 361 (52.6)	39 082 (53.3)	16 279 (50.8)	0.050	28 561 (51.4)	14 353 (51.6)	14 208 (51.1)	0.010
Male	49 933 (47.4)	34 198 (46.7)	15 735 (49.2)	0.050	27 057 (48.6)	13 274 (48.4)	13 601 (48.9)	0.010
Receiving public assistance	15 064 (14.3)	9178 (12.5)	5886 (18.4)	0.151^a^	9119 (16.4)	4551 (16.4)	4568 (16.4)	0.002
Elderly home residents	1374 (1.3)	418 (0.6)	956 (3.0)	0.142^a^	805 (1.4)	389 (1.4)	416 (1.5)	0.008
Clinical								
Duration of diabetes, mean (SD) y	5.84 (4.22)	5.80 (4.20)	5.91 (4.25)	0.026	5.90 (4.20)	5.91 (4.23)	5.89 (4.18)	0.004
Charlson comorbidity index score, mean (SD)^b^	0.81 (1.31)	0.59 (1.10)	1.33 (1.69)	0.435^a^	1.00 (1.38)	1.00 (1.46)	1.01 (1.30)	0.011
Hypertension diagnosis	80 441 (76.4)	55 566 (75.8)	24 875 (77.7)	0.045	42 951 (77.2)	21 431 (77.1)	21 520 (77.4)	0.008
Use of RAMP-hypertension service	500 (0.5)	458 (0.6%)	42 (0.1%)	0.136^a^	123 (0.2)	81 (0.3)	42 (0.2)	0.036
Any macrovascular events	3332 (3.2)	1349 (1.8)	1983 (6.2)	0.181^a^	2316 (4.2)	1037 (3.7)	1279 (4.6)	0.042
Any microvascular events	6013 (5.7)	2608 (3.6)	3405 (10.6)	0.230^a^	3819 (6.9)	1854 (6.7)	1965 (7.1)	0.016
Any hypoglycemic events	1493 (1.4)	692 (0.9)	801 (2.5)	0.100^a^	998 (1.8)	458 (1.6)	540 (1.9)	0.021
Laboratory tests, mean (SD)								
Hemoglobin A_1c_ level %	7.28 (1.32)	7.24 (1.25)	7.36 (1.49)	0.080	7.31 (1.37)	7.31 (1.33)	7.31 (1.41)	0.001
Fasting glucose, mg/dL	133.04 (40.36)	132.42 (37.86)	134.46 (45.59)	0.045	133.78 (42.52)	133.90 (41.32)	133.66 (43.63)	0.005
Body mass index^c^	25.4 (4.7)	25.5 (4.0)	25.3 (5.9)	0.034	25.3 (5.2)	25.4 (4.0)	25.3 (6.1)	0.006
Systolic blood pressure, mm Hg	136.1 (17.6)	134.9 (17.2)	139.0 (18.6)	0.221^a^	138.0 (18.0)	137.9 (18.0)	138.1 (18.0)	0.007
Diastolic blood pressure, mm Hg	73.9 (10.5)	74.1 (10.3)	73.4 (11.0)	0.069	73.6 (10.7)	73.7 (10.5)	73.6 (10.9)	0.011
Triglycerides, mg/dL	136.90 (94.69)	137.44 (95.41)	135.68 (94.25)	0.019	135.95 (92.03)	136.40 (88.53)	135.49 (95.29)	0.010
Low-density lipoprotein cholesterol, mg/dL	107.27 (32.05)	108.78 (31.18)	103.80 (33.68)	0.148^a^	105.56 (32.43)	105.81 (31.46)	105.31 (33.49)	0.015
Total cholesterol/high-density lipoprotein cholesterol ratio	3.93 (1.27)	3.95 (1.17)	3.89 (1.47)	0.038	3.90 (1.23)	3.91 (1.21)	3.89 (1.26)	0.012
Estimated glomerular filtration rate, mL/min/1.73 m^2^	99.1 (29.6)	103.7 (28.5)	88.5 (31.9)	0.476^a^	92.9 (29.4)	93.6 (28.0)	92.2 (30.8)	0.044
Medications								
Oral antidiabetic drugs	84 637 (80.4)	59 058 (80.6)	25 579 (79.9)	0.017	44 333 (79.7)	22 157 (79.)	22 176 (79.7%)	0.002
Antihypertensive drugs	87 606 (83.2)	59 422 (81.1)	28 184 (88.0)	0.214^a^	47 999 (86.3)	23 910 (86.0)	24 089 (86.6)	0.019
Oral lipid-lowering drugs	42 324 (40.2)	26 860 (36.7)	15 464 (48.3)	0.233^a^	25 198 (45.3)	12 540 (45.1)	12 658 (45.5)	0.009
Insulin use	8965 (8.5)	3795 (5.2)	5170 (16.1)	0.298^a^	6012 (10.8)	2996 (10.8)	3016 (10.8)	0.002

Abbreviations: ASMD, absolute standardized mean difference; RAMP-DM, Risk Assessment and Management Program-Diabetes Mellitus.

To convert fasting glucose to millimoles per liter, multiply by .0555; hemoglobin A1c to proportion of total hemoglobin, multiply by 0.01; low-density lipoprotein cholesterol to millimoles per liter, multiply by .0259; triglycerides to millimoles per liter, multiply by .0113.

^a^ ASMD greater than 0.100.

^b^ Charlson comorbidity index calculation did not include diabetes.

^c^ Body mass index was calculated as weight in kilograms divided by height in meters squared.

### Dementia Risk in RAMP-DM vs Usual Care Group

During follow-up (median [IQR] 8.4 [6.8-8.8] years), 1938 patients (6.97%) in the RAMP-DM group and 2728 patients (9.81%) in the usual care group received a diagnosis of dementia (Table 2). The mean (SD) age at the time of dementia diagnosis was 82.2 (7.3) years (median [IQR] age, 83 [78-87] years) for the RAMP-DM group and 82.6 (7.7) years (median [IQR] age, 83 [78-88] years) in the the usual care group. The incidence rate of dementia was 9.31 per 1000 person-years for the RAMP-DM and 14.02 per 1000 person-years for the usual care group. Estimated by type of dementia, the incidence rate per 1000 person-years for the RAMP-DM group was 2.43 for AD, 0.94 for VD, and 5.80 for other or unspecified dementia; in the usual care group, the incidence rate per 1000 person-years was was 3.13 for AD, 1.64 for VD, and 8.94 for other or unspecified dementia. The ARR was 2.84% (95% CI, 2.38%-3.30%) for all-cause dementia, 0.41% (95% CI, 0.17%-0.64%) for AD, 0.46% (95% CI, 0.30%-0.63%) for VD, and 1.97% (95% CI, 1.60%-2.35%) for other or unspecified dementia. The RRR was 29.0% (95% CI, 24.8%-32.8%) for all-cause dementia, 18.0% (95% CI, 7.9%-26.9%) for AD, 39.1% (95% CI, 27.5%-48.8%) VD, and 31.0% (95% CI, 25.9%-35.7%) for other or unspecified dementia (Table 3). Compared with the usual care group, the HbA_1C_ levels were consistently lower in the RAMP-DM group across the follow-up period (eTable 2 in Supplement 1). Compared with the usual care group, patients in the RAMP-DM group had a 28% lower risk of incidence of all-cause dementia (aHR, 0.72; 95% CI, 0.68-0.77; *P* < .001), 15% lower risk of AD (aHR, 0.85; 95% CI, 0.76-0.96; *P* = .009), 39% lower risk of VD (aHR, 0.61; 95% CI, 0.51-0.73; *P* < .001), and 29% lower risk of other or unspecified dementia (aHR, 0.71; 95% CI, 0.66-0.77; *P* < .001) (Figure and Table 3).

**Table 2.** Incidence of Dementia in RAMP-DM vs Usual Care Group

Dementia type	Cumulative incidence, No. of events (%) (n = 27 809 in each group)	Incidence rate per 1000 person-years	Person-years, No.	Follow-up duration, median (IQR) y
All-cause dementia				
Usual care group	2728 (9.81)	14.02	194 573	8.7 (5.3-8.9)
RAMP-DM group	1938 (6.97)	9.31	208 092	8.3 (8.0-8.6)
Alzheimer disease				
Usual care group	629 (2.26)	3.13	200 692	8.7 (6.2-8.9)
RAMP-DM group	516 (1.86)	2.43	212 186	8.4 (8.0-8.6)
Vascular dementia				
Usual care group	330 (1.19)	1.64	201 552	8.7 (6.3-8.9)
RAMP-DM group	201 (0.72)	0.94	213 120	8.4 (8.0-8.6)
Other or unspecified dementia				
Usual care group	1769 (6.36)	8.94	197 885	8.7 (5.8-8.9)
RAMP-DM group	1221 (4.39)	5.80	210 534	8.3 (8.0-8.6)

Abbreviation: RAMP-DM, Risk Assessment and Management Program-Diabetes Mellitus.

**Table 3.** Risk Reduction of Dementia Incidence Associated With Use of RAMP-DM Services

Dementia type	ARR (95% CI), %	RRR (95% CI), %	Univariate Cox regression estimation, crude HR (95% CI)	*P* value	Multiple Cox regression estimation, adjusted HR (95% CI)^a^	*P* value
All-cause dementia	2.84 (2.38-3.30)	29.0 (24.8-32.8)	0.66 (0.63-0.70)	<.001	0.72 (0.68-0.77)	<.001
Alzheimer disease	0.41 (0.17-0.64)	18.0 (7.9-26.9)	0.77 (0.69-0.87)	<.001	0.85 (0.76-0.96)	.009
Vascular dementia	0.46 (0.30-0.63)	39.1 (27.5-48.8)	0.57 (0.48-0.69)	<.001	0.61 (0.51-0.73)	<.001
Other or unspecified dementia	1.97 (1.60-2.35)	31.0 (25.9-35.7)	0.65 (0.60-0.70)	<.001	0.71 (0.66-0.77)	<.001

Abbreviations: ARR, absolute risk reduction; HR, hazard ratio; RAMP-DM, Risk Assessment and Management Program-Diabetes Mellitus; RRR, relative risk reduction.

^a^ The HR was calculated after adjusting for age at baseline; sex; recipient of public assistance; living in the elderly care home; duration of type 2 diabetes; hypertension diagnosis; Charlson Comorbidity Index; any hypoglycemic, macrovascular, and microvascular events; use of services in RAMP for patient with hypertension; hemoglobin A_1C_; fasting glucose level; body mass index; systolic and diastolic blood pressure; low-density lipoprotein cholesterol; total cholesterol to high-density lipoprotein cholesterol ratio; triglycerides; estimated glomerular filtration rate; and use of insulin, oral antidiabetic, antihypertensive, and lipid-lowering drugs. All baseline covariates were adjusted in the Cox regression.

**Figure.**
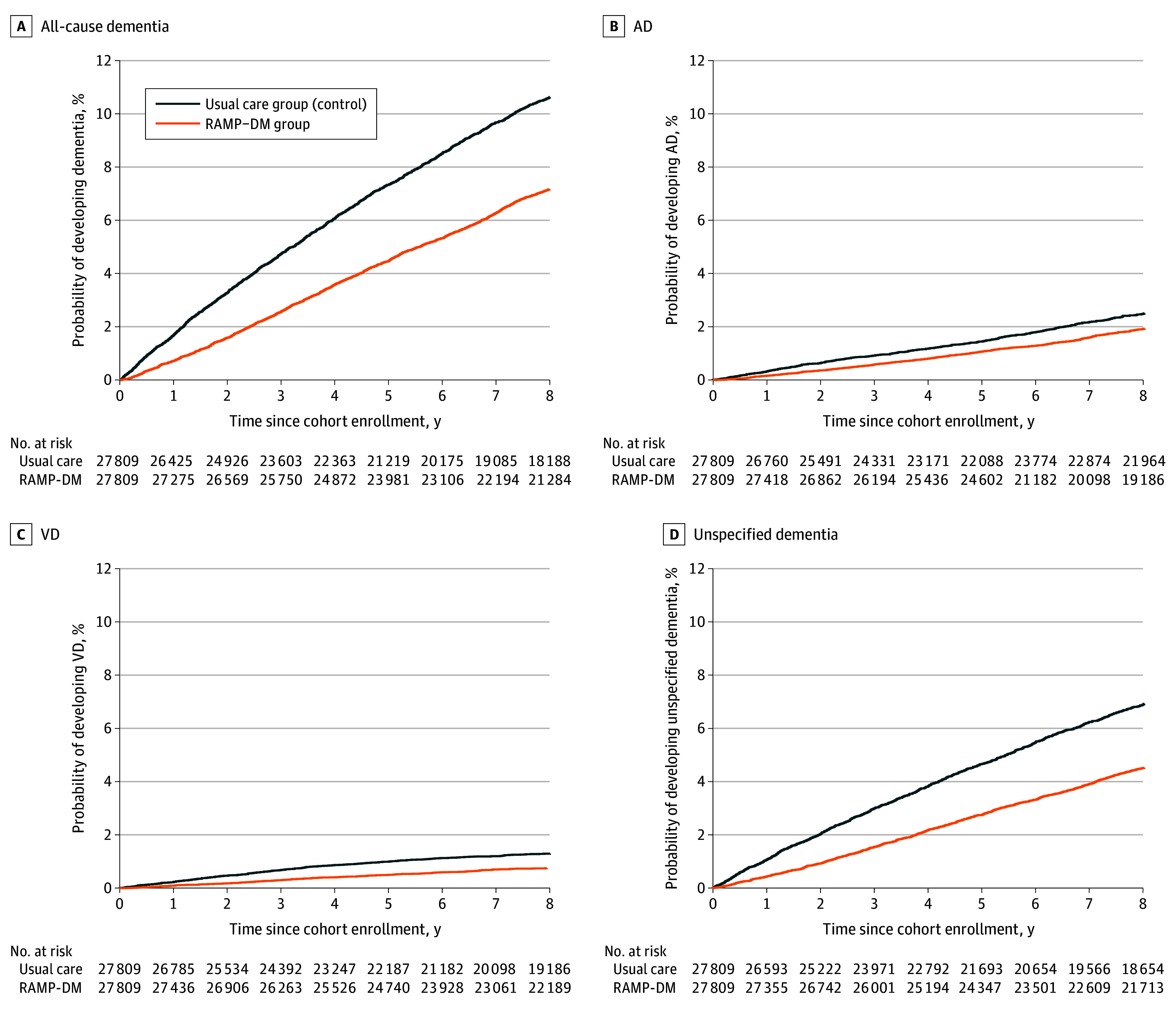
Time to Dementia Incidence in RAMP-DM vs Usual Care Group AD indicates Alzheimer disease; RAMP-DM, Risk Assessment and Management Program-Diabetes Mellitus; VD, vascular dementia.

### Sensitivity and Subgroup Analysis

Sensitivity analysis showed that the risk of dementia incidence was consistently lower in the RAMP-DM group compared with the usual care group in analysis with alternative criteria for patient inclusion or definition for the outcome (eTable 3 in Supplement 1). In subgroup analysis, the results showed that RAMP-DM attendance was associated with a risk reduction of dementia incidence in different subgroups (eTable 4 in Supplement 1). The association of RAMP-DM with dementia incidence was larger for patients with an HbA_1C_ level less than or equal to 7.5% and for those with hypertension (for incidence of vascular dementia), while there was no significant difference in the aHR estimates between subgroups based on sex, receipt of public assistance, elderly home residency, and CCI score (eTable 5 in Supplement 1).

### HbA_1C_ Level and Dementia Risk

HbA_1C_ level during follow-up was found to be associated with the risk of dementia incidence (Table 4, eTable 6, and eTable 7 in Supplement 1). A U-shape association was observed from the Cox model with the control of baseline covariates (eFigure 2 and eFigure 3 in Supplement 1). With reference to mean HbA_1C_ levels during first through third year after cohort entry (between 6.5%-7.5%), a higher risk of dementia incidence was found for patients with HbA_1C_ levels between 7.5% and 8.5% (aHR, 1.33; 95% CI, 1.19-1.48) and greater than 8.5% (aHR, 1.54; 95% CI, 1.31-1.80). For patients with HbA_1C_ levels lower than the reference group, the risk of dementia incidence was also higher (aHR for HbA_1C_ of 6%-6.5%, 1.17; 95% CI, 1.07-1.29; aHR for HbA_1C_ <6%, 1.39; 95% CI, 1.24-1.57).

**Table 4.** Association of Hemoglobin A_1c_ Level With Risk of Dementia Incidence Among RAMP-DM Participants

3-y mean hemoglobin A_1c_ level and dementia type	aHR (95% CI)	*P* value
≤6%		
All-cause dementia	1.39 (1.24-1.57)	<.001
Alzheimer disease	1.62 (1.24-2.12)	<.001
Vascular dementia	1.46 (0.99-2.15)	.06
Other or unspecified dementia	1.40 (1.20-1.63)	<.001
6-6.5%		
All-cause dementia	1.17 (1.07-1.29)	.001
Alzheimer disease	1.18 (0.94-1.47)	.15
Vascular dementia	1.11 (0.81-1.53)	.52
Other or unspecified dementia	1.18 (1.05-1.34)	.007
6.5-7.5%		
All-cause dementia	1 [Reference]	NA
Alzheimer disease	1 [Reference]	NA
Vascular dementia	1 [Reference]	NA
Other or unspecified dementia	1 [Reference]	NA
7.5-8.5%		
All-cause dementia	1.33 (1.19-1.48)	<.001
Alzheimer disease	1.14 (0.87-1.50)	.35
Vascular dementia	1.49 (1.06-2.09)	.02
Other or unspecified dementia	1.37 (1.19-1.57)	<.001
>8.5%		
All-cause dementia	1.54 (1.31-1.80)	<.001
Alzheimer disease	1.51 (1.02-2.22)	.04
Vascular dementia	1.98 (1.27-3.11)	.003
Other or unspecified dementia	1.66 (1.37-2.02)	<.001

Abbreviations: aHR, adjusted hazard ratio; NA, not applicable; RAMP-DM, Risk Assessment and Management Program-Diabetes Mellitus.

## Discussion

Although the association of T2D with dementia has been established in the literature, this, to our knowledge, is the first study to evaluate the outcomes of a multidisciplinary intervention program for T2D management to reduce the incidence of dementia. Our findings highlighted that diabetes management in the primary care setting is prospectively associated with a reduced risk of subsequent dementia incidence and strengthened the evidence on the associations of T2D with the incidence of different dementia subtypes. Our study also identified a U-shaped association of HbA_1C_ levels with dementia incidence risk among patients enrolled in RAMP-DM.

The study outcome showed that patients in RAMP-DM had a 28% lower risk for all-cause dementia incidence. Compared with literature from a previous meta-analysis^4,5,30,31,32^ that showed the risk ratio between T2D and dementia ranged from 1.33 to 1.62, patients in RAMP-DM had a substantially lower risk for dementia but they were still at slightly higher risk than those without T2D. This result is consistent with the evidence from previous studies^12,13,14,15^ showing that glycemic control is associated with the risk of dementia among patients with T2D, given that the HbA_1C_ level was found to be consistently lower in the RAMP-DM group than that in the usual care group during follow-up. Previous studies^24,26^ reported that RAMP-DM was likely to enable better management for patients with diabetes in terms of better control of blood glucose and reduced risks of diabetic complications. A study of a large cohort in the UK^13^ found that microvascular complication was associated with a 10% higher hazard of developing dementia, particularly for neuropathy (25% higher hazard) and nephropathy (23% higher hazard). It also demonstrated that an elevated HbA_1C_ level was associated with a higher risk of dementia incidence.^13^ In a Swedish study using national registry data,^15^ patients with HbA_1C_ more than 76 mmol/mol (9.1%) had a higher risk of AD and patients with HbA_1C_ more than 53 mmol/mol (7.0%) had a higher risk for VD, compared with those with an HbA_1C_ less than 52 mmol/mol (6.9%). For diabetic complications, studies have found that nephropathy,^33^ diabetic retinopathy,^34,35^ and cardiovascular complications^36,37,38^ are associated with a higher risk of dementia. In interventional studies, a systematic review^39^ reported that the use of metformin is associated with lower risks for cognitive impairment (odds ratio = 0.55) and dementia (HR = 0.76), whereas the risk of dementia or cognitive impairment for patients with T2D in multifactorial interventions has not been reported in existing studies.^16,19,20,21^

This study also found that risk reduction for VD (39% reduction) was higher than AD (15% reduction) in patients receiving RAMP-DM vs usual care. This aligns with the findings from several meta-analyses^30,32,40^ that the risk for VD was higher than nonvascular dementia (RR for women, 2.34 for VD vs 1.53 for non-VD; RR for men, 1.73 for VD vs 1.49 for non-VD) or AD (RR for VD, 2.48 vs RR for AD, 1.46). Because T2D is associated with increased risk of stroke and other macrovascular complications, it is not surprising to find out that RAMP-DM was associated with lower VD incidence. Further studies can be conducted to reveal the underlying mechanisms that contribute to the development of different types of dementia among patients with T2D.

It was also revealed that the 3-year mean HbA_1C_ levels that were 6.5% or less and greater than 7.5% were both associated with greater risk for dementia. HbA_1C_ levels of 6.5% or lower were considered a strict treatment target for patients with T2D, with 6.5% to 7.5% being the intermediate or lenient target.^29^ Although the 3-year mean HbA_1C_ level after cohort enrollment represented a mean blood glucose level rather than actual glucose levels that may vary across individuals, this association of HbA_1C_ level with risk of dementia strengthened the evidence that poor glycemic control may be a factor underlying the association with dementia incidence, which is in line with a previous cohort study^41^ that found a U-shaped association of glucose levels with the risk of dementia. Furthermore, although the association of hyperglycemia with dementia has been studied (as mentioned earlier), the association of dementia with lower HbA_1C_ levels may be attributed to the presence of hypoglycemic events because there is a greater risk for hypoglycemia among patients with T2D and HbA_1C_ levels less than 6%.^42^ In the literature, a history of hypoglycemic episodes was found to be associated with a greater risk of dementia.^43^ The UK cohort study^13^ also found that larger HbA_1C_ variation and the presence of hypoglycemic events were associated with the risk of all-cause dementia as well. A study in Hong Kong^44^ showed that patients with HbA_1C_ levels greater than 7.5% at baseline and decreased their levels to less than 7.5% in 1 year, had a higher risk of dementia. Additionally, an RCT^45,46^ found that patients with intensive glycemic control (HbA_1C_ target at <6.0%) did not have cognitive function improvement vs those with standard glycemic control (HbA_1C_ target at 7.0%-7.9%). This RCT study^47^ along with the aforementioned one^45,46^ suggested that there is no evidence to set an HbA_1C_ treatment target of less than 6.0% to reduce the risk of developing dementia, which aligns with the American Diabetes Association recommendations that glycemic control target should be less than 7% for most patients and less than 7.5% or 8% to 8.5% for older persons with different comorbidities.

The neurological benefits of RAMP-DM may result from its risk stratification process and multidisciplinary coordination.^24,26^ Risk stratification in RAMP-DM can prioritize patients’ needs for diabetes management based on their risk level and may potentially enable more individualized management of glycemic levels that may reduce the risk of hypoglycemic events. Patients can be followed up regularly by nurses and other health care professionals under the multidisciplinary framework. Their clinical needs can then be identified and addressed in a timely manner through referral to different health care professionals. The patients were also empowered for T2D management by health education to facilitate their own T2D management and self-care. In addition, RAMP-DM may enable modifications on several factors associated with T2D risk, such as obesity and physical inactivity, which are also associated with increased risk of dementia independent from T2D.^48^

### Limitations

This study includes several limitations. First, selection bias existed as in other studies using EHR data. Because HADCL only includes health records within the public health care system, attendance records in the private sector for T2D management were not captured; however, HADCL is the largest and most representative database available to comprehensively assess the health conditions of the Hong Kong population. In addition, although we have adopted propensity score matching using multidimensional baseline characteristics to reduce the bias and make the 2 groups comparable, patients in the RAMP-DM group were still likely to have greater health consciousness than those in the usual care group, which may have lead to differences in dementia incidence. This association of the use of RAMP-DM with unmeasured baseline characteristics in EHRs such as health literacy, attitudes toward glycemic control, and cognitive functioning level, may influence the estimated association of the use of RAMP-DM with dementia incidence. We have conducted a sensitivity analysis on a subsample without dementia incidence or death in the first 2 years following the index date to reduce the likelihood of reverse causality, where people with dementia or worse health conditions may have been less likely to join RAMP-DM in addition to usual care. In the future, these unmeasured baseline characteristics that are likely associated with the incidence of dementia should be adjusted in prospective cohort or experimental settings. Second, there were information biases, including undiagnosed and unspecified types of dementia, which may have lead to the underestimation of the incidence of all-cause and subtype of dementia.^49^ Assuming misclassification of dementia was nondifferential between the 2 studied groups, our outcome may have underestimated the ARR for dementia.^50^ Limited information on the duration of individuals’ stay in the RAMP-DM program and referral records from nurses or general physicians to specific specialists or allied health services (eg, dietitians) may have also hindered in-depth analysis of the association of these characteristics with the outcomes. Third, because the smoking status of patients in the usual care group who did not have respiratory conditions was rarely found in the database, it was not included as a covariate. The smoking rate among patients with T2D was low and similar between patients using RAMP-DM and those who were not (11.2% vs 11.8%),^24^ which suggested that it was not very likely that smoking status would alter the association of RAMP-DM with dementia incidence. We also matched and controlled various characteristics associated with smoking, including the presence of coronary heart disease (in macrovascular events), chronic obstructive pulmonary disease, and cancers (in CCI). Last, our results should be interpreted with caution. Although an HbA_1C_ level of 6.5% to 7.5% was found to be associated with a lower incidence of dementia in the supplementary analysis, it should not be interpreted as an optimal glycemic control level, particularly for health outcomes other than cognitive functions. For outcomes of dementia incidence, the glycemic control targets may be different across age groups of patients with T2D, which is subject to further studies. Moreover, as a common limitation in observational studies, our findings should not be interpreted as indicating causality.

## Conclusions

This cohort study of patients with T2D provided evidence that a multidisciplinary and individualized diabetes management program in primary care settings was beneficial to patients with T2D in lowering the likelihood of all-cause dementia and its major subtypes. The association of HbA_1C_ level with dementia risk suggested the contribution of poor glycemic control to dementia incidence, where glycemic control that was either too stringent (HbA_1C_ ≤ 6.5%) or not meeting the control target (HbA_1C _> 7.5%) was associated with an increased risk of dementia. Further studies could make use of prospective cohorts or RCTs to verify the effectiveness of similar diabetic management interventions on dementia risks, and investigate the biological mechanism of such effects. The cost-effectiveness of such a program for reducing the risk of dementia incidence also requires further evaluation.

## Notes

Supplement 1. **eTable 1.** Codes Used to Define the Diagnoses **eTable 2.** Comparison of Baseline Characteristics Between Patients With and Without Missing Values **eFigure 1.** Flow Chart for Inclusion and Exclusion of Patients **eTable 3.** Trends of HbA_1C_ Level of Patients in RAMP-DM vs Usual Care Group During Follow-Up **eTable 4.** Sensitivity Analysis for RAMP-DM on Dementia Incidence **eTable 5.** Subgroup Analysis for RAMP-DM on Dementia Incidence **eTable 6.** Association Between HbA_1C_ Level During 1st-3rd Year After Cohort Entry and Risk of Dementia Incidence After 3rd Year Among RAMP-DM Participants **eTable 7.** Association Between HbA_1C_ Level During 4th-6th Year After Cohort Entry and Risk of Dementia Incidence Among RAMP-DM Participants **eFigure 2.** Adjusted Hazard Ratio for Dementia Risk by Different HbA_1C_ Level During Follow-Up **eFigure 3.** Kaplan-Meier Curve for Dementia Incidence by Different HbA_1C_ Level During Follow-Up

Supplement 2.
Data Sharing Statement
