# Supplementary material for: Risk of Dementia Among Patients With Diabetes in a Multidisciplinary, Primary Care Management Program
Source: JAMA Netw Open. 2024 Feb 12;7(2):e2355733. doi: 10.1001/jamanetworkopen.2023.55733 (PMC10862158; doi:10.1001/jamanetworkopen.2023.55733)
Supplement: Supplement 1. — eTable 1. Codes Used to Define the Diagnoses eTable 2. Comparison of Baseline Characteristics Between Patients With and Without Missing Values eFigure 1. Flow Chart for Inclusion and Exclusion of Patients eTable 3. Trends of HbA1C Level of Patients in RAMP-DM vs Usual Care Group During Follow-Up eTable 4. Sensitivity Analysis for RAMP-DM on Dementia Incidence eTable 5. Subgroup Analysis for RAMP-DM on Dementia Incidence eTable 6. Association Between HbA1C Level During 1st-3rd Year After Cohort Entry and Risk of Dementia Incidence After 3rd Year Among RAMP-DM Participants eTable 7. Association Between HbA1C Level During 4th-6th Year After Cohort Entry and Risk of Dementia Incidence Among RAMP-DM Participants eFigure 2. Adjusted Hazard Ratio for Dementia Risk by Different HbA1C Level During Follow-Up eFigure 3. Kaplan-Meier Curve for Dementia Incidence by Different HbA1C Level During Follow-Up [file jamanetwopen-e2355733-s001.pdf]

Supplemental Online Content

Wang K, Zhao S, Lee EKP, et al. Risk of dementia among patients with diabetes in a multidisciplinary, primary care management program. *JAMA Netw Open*. 2024;7(2):e2355733. doi:10.1001/jamanetworkopen.2023.55733

eTable 1. Codes Used to Define the Diagnoses
eTable 2. Comparison of Baseline Characteristics Between Patients With and Without Missing Values
eFigure 1. Flow Chart for Inclusion and Exclusion of Patients
eTable 3. Trends of HbA_{1C} Level of Patients in RAMP-DM vs Usual Care Group During Follow-Up
eTable 4. Sensitivity Analysis for RAMP-DM on Dementia Incidence 5. Subgroup Analysis for RAMP-DM on Dementia Incidence
eTable 6. Association Between HbA_{1C} Level During 1st-3rd Year After Cohort Entry and Risk of Dementia Incidence After 3rd Year Among RAMP-DM Participants
eTable 7. Association Between HbA_{1C} Level During 4th-6th Year After Cohort Entry and Risk of Dementia Incidence Among RAMP-DM Participants
eFigure 2. Adjusted Hazard Ratio for Dementia Risk by Different HbA_{1C} Level During Follow-Up
eFigure 3. Kaplan-Meier Curve for Dementia Incidence by Different HbA_{1C} Level During Follow-Up

This supplemental material has been provided by the authors to give readers additional information about their work.

© 2024 Wang K et al. *JAMA Network Open*.

eTable 1. Codes Used to Define the Diagnoses

Diagnoses	ICD-10	ICPC-2
Type 2 diabetes	E11	T90
Type 1 diabetes	E10	T89
Gestational diabetes	O24	W85
All-cause dementia	F00, F01, F02.0, F02.2, F02.3, F02.8, F03, F05.1, G30, G31, I67.3	P70
Alzheimer's disease	F00, G30	-
Vascular dementia	F01, I67.3	-
Other & unspecified dementia	F02.0, F02.2, F02.3, F02.8, F03, F05.1, G31	P70
Hypoglycemia	E16.0-E16.2	T87
Macrovascular events	I25, I63, I70, I73.9, E11.5	K74-K76, K89-K92
Microvascular events	E11.2, E11.3, E11.4, G57, G67.2	N94, F83
Hypertension	I10-I13, I15	K86, K87

Note: ICD-10-CM, International Classification of Diseases, 10th revision, Clinical Modification; ICPC-2, International Classification of Primary Care, 2nd edition. ICPC-2 codes were used to identify diagnoses recorded in family medicine datasets. For diagnoses recorded for the other services, an in-house coding system were used in Hospital Authority Data Collaboration Lab (HADCL) databases, namely term ID. One ICD-10 code (with one letter and three digits) is matched to one or multiple term IDs. The diagnoses used in this study is defined in ICD-10 codes, and selected based on a mapping table provided by HADCL that links ICD-10 codes to term IDs.

© 2024 Wang K et al. *JAMA Network Open*.

eTable 2. Comparison of Baseline Characteristics Between Patients With and Without Missing Values

	Unmatched patients	
	Without missing values	With missing values
No. of patients	105,294	12,287
Socio-demographics		
Age at baseline (year)	66.13 (11.89)	68.62 (13.14)
Female	49933 (47.4%)	5609 (45.6%)
Male	55361 (52.6%)	6678 (54.4%)
With public assistance	15064 (14.3%)	2088 (17.0%)
Elderly home residents	1374 (1.3%)	598 (4.9%)
Clinical characterstics		
Duration of diabetes (year)	5.84 (4.22)	5.29 (4.24)
Charlson comorbidity index	0.81 (1.35)	0.98 (1.67)
Hypertension diagnosis	14410 (73.3%)	75343 (76.9%)
Use of RAMP-HT service	67 (0.3%)	458 (0.5%)
Any macrovascular events	549 (2.8%)	3311 (3.4%)
Any microvascular events	1015 (5.2%)	5867 (6.0%)
Hypoglycemic events	314 (1.6%)	1492 (1.5%)
Laboratory tests		
HbA1c (%)	7.28 (1.32)	7.28 (1.37)
Fasting glucose (mmol/L)	7.38 (2.24)	7.32 (2.73)
BMI (kg/m^2)	25.4 (4.7)	25.2 (4.2)
SBP (mmHg)	136.14 (17.73)	135.63 (18.11)
DBP (mmHg)	73.91 (10.56)	73.42 (10.95)
Triglycerides (mmol/L)	1.55 (1.07)	1.55 (1.33)
LDL cholestrol (mmol/L)	2.78 (0.83)	2.74 (0.84)
TC/HDL-C ratio	3.93 (1.27)	3.94 (1.26)
eGFR (ml/min/1.73m^2)	99.11 (30.37)	96.03 (33.01)
Medications		
Oral antidiabetic drugs	84637 (80.4%)	9517 (77.5%)
Antihypertensive drugs	87606 (83.2%)	10169 (82.8%)
Oral lipid-lowering drugs	42324 (40.2%)	4167 (33.9%)
Insulin use	8965 (8.5%)	1179 (9.6%)

Note: Statistics in the table are Mean (SD) or N (%).

© 2024 Wang K et al. *JAMA Network Open*.

eFigure 1. Flow chart for Inclusion and Exclusion of Patients

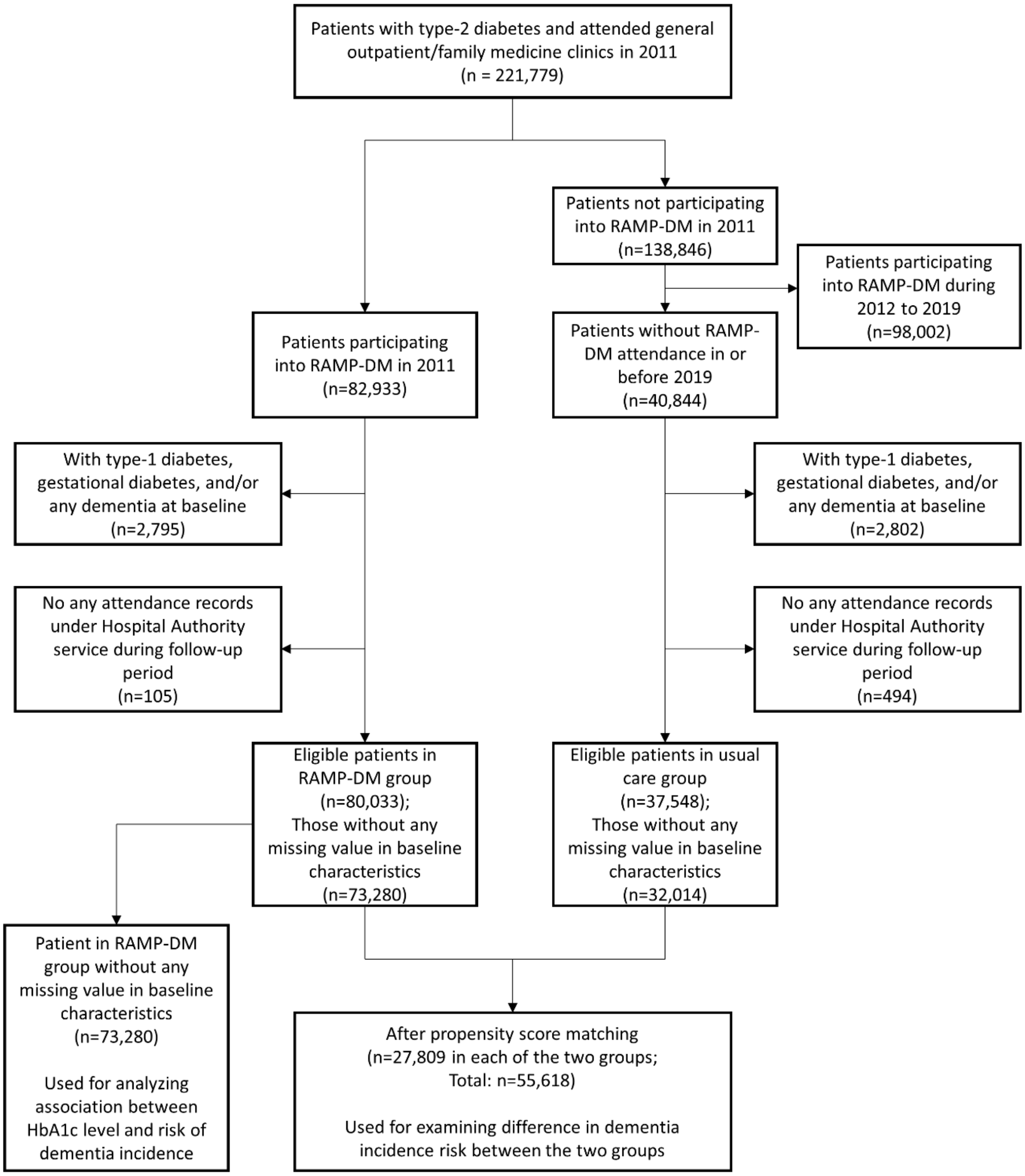

© 2024 Wang K et al. *JAMA Network Open*.

eTable 3. Trends of HbA_{1c} Level of Patients in RAMP-DM vs Usual Care Group During Follow-Up

Number of year during follow-up	RAMP-DM group	Usual care group	ASMD	P value
Mean HbA1c level (%)				
Year 1	7.18±1.08	7.28±1.25	0.086	<0.001
Year 2	7.09±1.07	7.29±1.26	0.171	<0.001
Year 3	7.08±1.11	7.35±1.32	0.221	<0.001
Year 4	7.09±1.12	7.27±1.29	0.149	<0.001
Year 5	7.16±1.16	7.30±1.27	0.115	<0.001
Year 6	7.19±1.17	7.33±1.29	0.114	<0.001
Year 7	7.22±1.18	7.32±1.28	0.081	<0.001
Year 8	7.28±1.19	7.34±1.26	0.049	<0.001
Percentage of patients with mean HbA1c >= 7%				
Year 1	51.1%	52.7%	0.033	<0.001
Year 2	46.0%	52.4%	0.128	<0.001
Year 3	44.9%	54.3%	0.189	<0.001
Year 4	45.4%	51.6%	0.124	<0.001
Year 5	48.2%	53.2%	0.099	<0.001
Year 6	48.8%	54.2%	0.110	<0.001
Year 7	50.5%	54.4%	0.079	<0.001
Year 8	53.3%	56.3%	0.060	<0.001
Percentage of patients with mean HbA1c >= 8%				
Year 1	17.5%	21.1%	0.090	<0.001
Year 2	15.4%	21.4%	0.155	<0.001
Year 3	15.3%	23.1%	0.200	<0.001
Year 4	15.6%	22.0%	0.165	<0.001
Year 5	17.5%	22.4%	0.122	<0.001
Year 6	17.7%	22.9%	0.129	<0.001
Year 7	18.4%	22.5%	0.102	<0.001
Year 8	19.8%	22.7%	0.072	<0.001

Note: ASMD, absolute standardized mean difference. Statistics in the table are mean ± SD. P values derived from independent sample t test.

© 2024 Wang K et al. *JAMA Network Open*.

eTable 4. Sensitivity Analysis for RAMP-DM on Dementia Incidence

Scenarios	All-cause dementia		AD		VD		Other/unspecified dementia	
	aHR	95%CI	aHR	95%CI	aHR	95%CI	aHR	95%CI
Sample without propensity score matching	0.71*	(0.67-0.74)	0.87*	(0.76-1.00)	0.57*	(0.49-0.66)	0.71*	(0.67-0.76)
Adjusting additional covariates in the model^	0.73*	(0.69-0.78)	0.86*	(0.77-0.97)	0.62*	(0.52-0.74)	0.72*	(0.67-0.78)
Excluding dementia or death within 2 years after cohort entry	0.75*	(0.70-0.80)	0.85*	(0.74-0.97)	0.67*	(0.55-0.83)	0.73*	(0.67-0.80)
Patients aged 60 years or above	0.72*	(0.67-0.76)	0.85*	(0.75-0.95)	0.59*	(0.49-0.70)	0.71*	(0.66-0.76)
Excluding patients died during follow-up	0.79*	(0.73-0.86)	0.91	(0.78-1.06)	0.72*	(0.55-0.94)	0.77*	(0.69-0.86)
Excluding patients died under 80 years during follow-up	0.75*	(0.71-0.80)	0.90	(0.80-1.02)	0.64*	(0.53-0.77)	0.73*	(0.68-0.79)

Note: *P<0.05. ^The covariates included whether used outpatient service in past 1 year, whether visited emergency room in past 1 year, whether hospitalized in past 3 years, and presence of hyperglycemic events (diabetic ketoacidosis and hyperosmolar hyperglycemic state) prior to index date. AD, Alzheimer's disease; VD, vascular dementia; aHR, adjusted hazard ratio; CI, confidence interval.

© 2024 Wang K et al. *JAMA Network Open*.

eTable 5. Subgroup Analysis for RAMP-DM on Dementia Incidence

Subgroups	All-cause dementia		AD		VD		Other/unspecified dementia	
	aHR	95%CI	aHR	95%CI	aHR	95%CI	aHR	95%CI
Male	0.67*	(0.61-0.74)	0.78*	(0.62-0.97)	0.52*	(0.39-0.68)	0.69*	(0.69-0.78)
Female	0.75*	(0.70-0.81)	0.88	(0.77-1.02)	0.69*	(0.55-0.87)	0.72*	(0.66-0.80)
With public assistance	0.73*	(0.68-0.78)	0.87*	(0.76-0.99)	0.65*	(0.53-0.80)	0.71*	(0.65-0.77)
No public assistance	0.69*	(0.61-0.78)	0.79	(0.59-1.06)	0.51*	(0.36-0.72)	0.72*	(0.62-0.84)
Elderly home	0.72*	(0.68-0.76)	0.86*	(0.76-0.97)	0.61*	(0.51-0.73)	0.71*	(0.65-0.76)
Not in elderly home	0.73*	(0.54-0.99)	0.75	(0.38-1.49)	0.61	(0.26-1.42)	0.81	(0.56-1.18)
With hypertension	0.68*	(0.57-0.81)	1.09	(0.79-1.50)	0.32*	(0.17-0.59)	0.62*	(0.50-0.78)
No hypertension	0.73*	(0.68-0.77)	0.82*	(0.72-0.93)	0.65*	(0.54-0.78)	0.73*	(0.67-0.78)
HbA1c: ≤6.5%	0.66*	(0.60-0.73)	0.77*	(0.63-0.94)	0.61*	(0.44-0.85)	0.65*	(0.57-0.74)
HbA1c: 6.5-7.5%	0.69*	(0.63-0.76)	0.87	(0.73-1.05)	0.52*	(0.39-0.69)	0.68*	(0.60-0.77)
HbA1c: >7.5%	0.86*	(0.77-0.97)	0.92	(0.72-1.17)	0.72*	(0.52-1.00)	0.88	(0.76-1.02)
CCI score: 0	0.68*	(0.65-0.76)	0.78*	(0.65-0.94)	0.64*	(0.47-0.87)	0.67*	(0.59-0.76)
CCI score: 1-2	0.76*	(0.69-0.82)	0.97	(0.81-1.15)	0.51*	(0.38-0.67)	0.75*	(0.67-0.83)
CCI score: 3+	0.79*	(0.68-0.92)	0.81	(0.59-1.13)	0.87	(0.61-1.25)	0.78*	(0.65-0.94)

Note: *P<0.05. AD, Alzheimer’s disease; VD, vascular dementia; aHR, adjusted hazard ratio; CI, confidence interval; CCI, Charlson comorbidity index.

© 2024 Wang K et al. *JAMA Network Open*.

eTable 6. Association Between HbA_{1c} Level During 1st-3rd Year After Cohort Entry and Risk of Dementia Incidence After 3rd Year Among RAMP-DM Participants

Mean HbA1c in 1st-3rd year	All-cause dementia		Alzheimer's disease		Vascular dementia		Unspecified dementia	
	aHR	95%CI	aHR	95%CI	aHR	95%CI	aHR	95%CI
≤6%	1.31*	(1.13, 1.52)	1.62*	(1.15, 2.29)	1.18	(0.69, 2.00)	1.33*	(1.10, 1.59)
6 - 6.5%	1.22*	(1.09, 1.36)	1.09	(0.81, 1.45)	1.20	(0.82, 1.76)	1.23*	(1.07, 1.42)
6.5 - 7.5% (reference)								
7.5 - 8.5%	1.39*	(1.22, 1.58)	1.30	(0.92, 1.82)	1.50*	(1.00, 2.24)	1.40*	(1.19, 1.64)
>8.5%	1.51*	(1.24, 1.82)	1.50	(0.89, 2.51)	1.70*	(0.98, 2.96)	1.68*	(1.33, 2.13)

Note: *P<0.05. Patients with dementia diagnosis or death record during 1st-3rd year after index date were excluded

© 2024 Wang K et al. *JAMA Network Open*.

eTable 7. Association Between HbA_{1c} Level During 4th-6th Year After Cohort Entry and Risk of Dementia Incidence Among RAMP-DM Participants

Three-year mean HbA1c during 4th-6th year after index date	All-cause dementia		Alzheimer's disease		Vascular dementia		Other/unspecified dementia	
	aHR	95%CI	aHR	95%CI	aHR	95%CI	aHR	95%CI
≤6%	1.61*	(1.39-1.86)	1.63*	(1.24-2.13)	2.03*	(1.29-3.20)	1.51*	(1.26-1.82)
6 - 6.5%	1.23*	(1.09-1.40)	1.22	(0.97-1.54)	0.76	(0.47-1.23)	1.30*	(1.11-1.51)
6.5 - 7.5% (reference)								
7.5 - 8.5%	1.17*	(1.01-1.36)	1.23	(0.93-1.62)	1.06	(0.66-1.71)	1.16	(0.96-1.40)
>8.5%	1.73*	(1.45-2.07)	1.74*	(1.24-2.44)	1.91*	(1.15-3.17)	1.65*	(1.31-2.07)

Note: *P<0.05. Patients with dementia diagnosis or death record during 1st-3rd year after index date were excluded

© 2024 Wang K et al. *JAMA Network Open*.

eFigure 2. Adjusted Hazard Ratio for Dementia Risk by Different HbA1C Level During Follow-Up

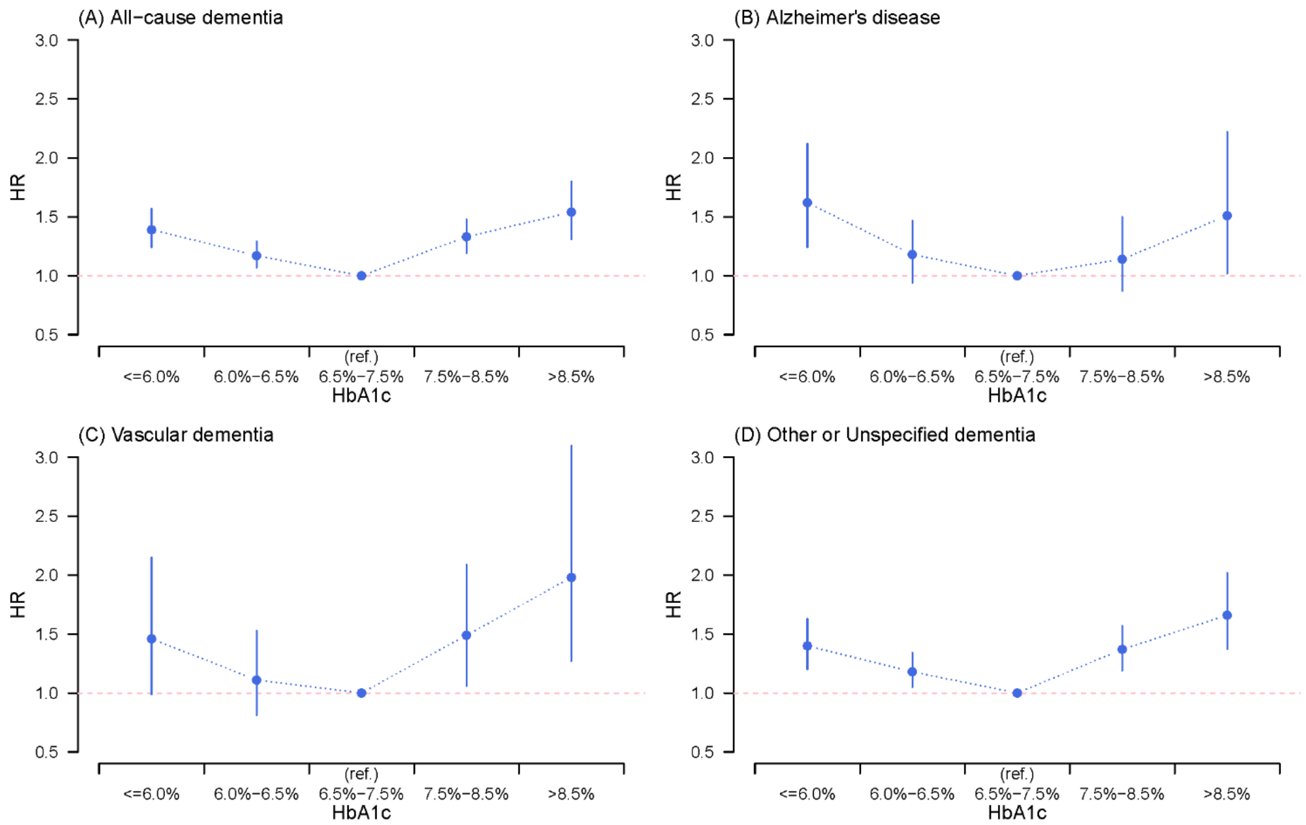

Note: HR, Hazard ratio. Error bars refer to 95% confidence interval. HbA1c level refers to the mean HbA1c level during the 1st-3rd year after index date.

© 2024 Wang K et al. *JAMA Network Open*.

eFigure 3. Kaplan-Meier Curve for Dementia Incidence By Different HbA1C Level During Follow-Up

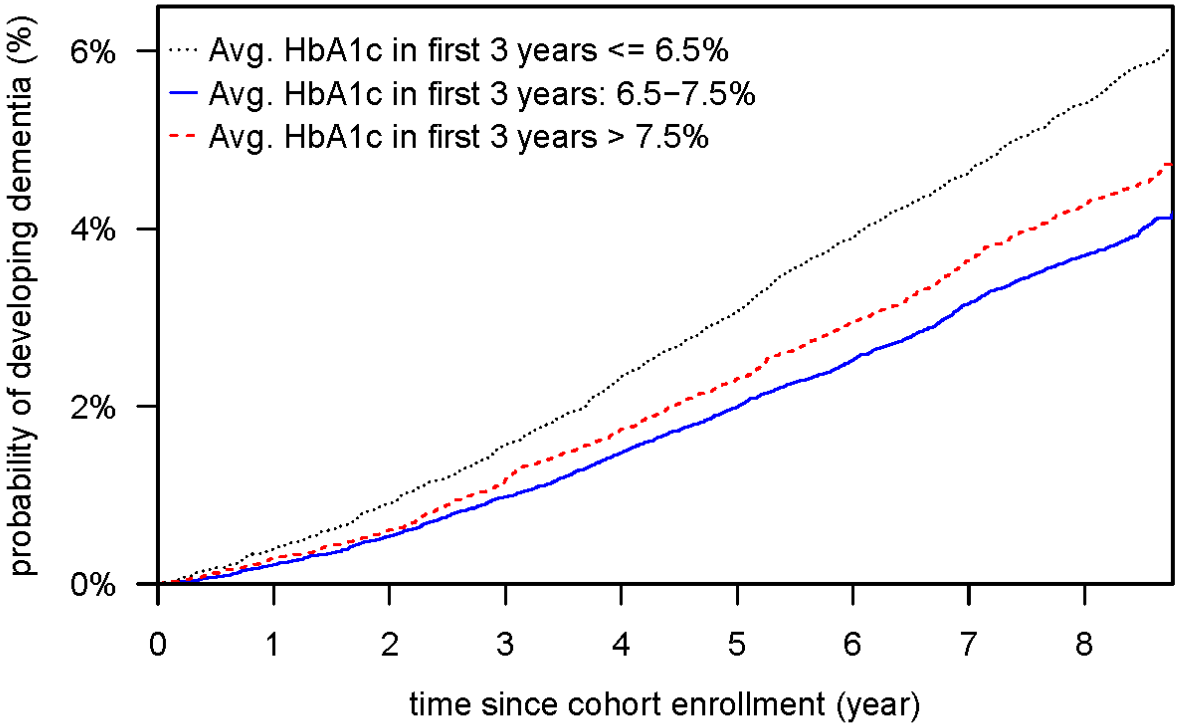

Note: HbA1c level refers to the mean HbA1c level during the 1st-3rd year after index date.

© 2024 Wang K et al. *JAMA Network Open.*
