# Supplementary material for: Risk of Dementia Among Patients With Diabetes in a Multidisciplinary, Primary Care Management Program
Source: JAMA Netw Open. 2024 Feb 12;7(2):e2355733. doi: 10.1001/jamanetworkopen.2023.55733 (PMC10862158; doi:10.1001/jamanetworkopen.2023.55733)
Supplement: Supplement 2. — Data Sharing Statement [file jamanetwopen-e2355733-s002.pdf]

Data Sharing Statement

Wang. Risk of Dementia Among Patients With Diabetes in a Multidisciplinary, Primary Care Management Program. *JAMA Netw Open*. Published February 12, 2024. doi:10.1001/jamanetworkopen.2023.55733

Data
Data available: No

Additional Information
Explanation for why data not available: The data will not be shared due to confidentiality in compliance with the policy of Hospital Authority Data Collaboration Lab (HADCL) released by Hospital Authority, Hong Kong Special Administrative Regions of China.
